# PBK/TOPK mediates geranylgeranylation signaling for breast cancer cell proliferation

**DOI:** 10.1186/s12935-015-0178-0

**Published:** 2015-02-28

**Authors:** Xiaoyan Dou, Jing Wei, Aiqin Sun, Genbao Shao, Chandra Childress, Wannian Yang, Qiong Lin

**Affiliations:** School of Medical Sciences and Laboratory Medicine, Jiangsu University, 301 Xuefu Road, Zhenjiang, Jiangsu China; Weis Center for Research, Geisinger Clinic, 100 N. Academy Avenue, Danville, PA17822 USA

**Keywords:** Breast cancer, Atorvastatin, PBK/TOPK, Geranylgeranylation, YAP

## Abstract

PDZ binding-kinase (PBK) (also named T-lymphokine-activated killer cell-originated protein kinase (TOPK)), a serine/threonine kinase, is tightly controlled in normal tissues but elevated in many tumors, and functions in tumorigenesis and metastasis. However, the signaling that regulates expression of PBK in cancer cells remains elusive. Here we show that atorvastatin (Lipitor), an inhibitor of hydroxymethylglutaryl co-enzyme A (HMG-CoA) reductase that is a rate-limiting enzyme of mevalonate pathway, down-regulates expression of PBK by impairing protein geranylgeranylation. The shRNA knockdown demonstrated that Yes-associated protein (YAP) mediates geranylgeranylation-regulated expression of PBK. Importantly, atorvastatin or the geranylgeranyltransferase I inhibitor GGTI-298 inhibited breast cancer cell proliferation through inactivation of YAP signaling and down-regulation of PBK. These findings have defined a new signaling pathway that regulated expression of PBK and identified PBK as a downstream target of the Hippo-YAP signaling, uncoverd a mechanism underlying the anti-cancer effect by inhibition of mevalonate pathway and geranylgeranylation, and provided a potential target for breast cancer targeted therapy.

## Background

Breast cancer is the most frequently diagnosed cancer and the leading cause of cancer death in females worldwide. Unraveling the molecular and cellular mechanisms underlying breast cancer progression and metastasis is necessary for development of therapeutic agents to treat this disease.

PDZ-binding kinase (PBK) (also named T-lymphokine-activated killer cell–originated protein kinase (TOPK)) is a 322 amino acid MAPKK-like serine/threonine protein kinase that was identified as an interleukin-2 induced gene in T-lymphokine-activated killer cells and as an interaction partner with the human tumor suppressor hDlg [1]. PBK is overexpressed in multiple types of cancer, including breast, prostate, colon, bladder, and lung cancer, but is undetectable in normal tissues except germ cells in the testis and several fetal tissues [2-5]. PBK mediates UVB-induced JNK activation and facilitates H-Ras-induced cell transformation [6]. In addition, PBK serves as an oncogenic kinase that exerts positive feedback on ERK2 to promote colorectal cancer formation *in vitro* and *in vivo* [7]. Furthermore, PBK physically interacts with the DBD domain of p53 and regulates the tumor suppressor function of p53 [8]. PBK stimulates AKT-dependent cell migration/invasion by relieving the PTEN-dependent suppressive effect, indicating its crucial role in cancer metastasis [9]. PBK phosphorylates histone H3 at Ser10 *in vitro* and *in vivo*, and to function as molecular marker in breast cancer [10]. Taken together, overexpression of PBK is correlated with oncogenesis.

Statins are a class of specific inhibitors of HMG-CoA reductase, a rate-limiting enzyme in the mevalonate pathway [11]. The mevalonate pathway is biologically important because the metabolites of the mevalonate pathway play vital roles in protein post-translational modifications such as geranylgeranylation and farnesylation, cell membrane integrity, respiration electronic chain reaction, and cholesterol synthesis [12]. As potent blockers of biosynthesis of cholesterol, statins have long been used in clinic to treat hypercholesterolemia and prevent cardiovascular diseases [12]. Because cellular function of many cell growth signaling molecules, particularly small GTPases, is dependent on prenylation that is blocked by statins, treatment of cancer cells with statins inhibits cell proliferative, migration, and invasion, and induces apoptosis. Thus, statins become a promising cancer therapeutic agent against many types of cancers, including breast cancer [12,13]. It has been demonstrated that statins trigger tumor-specific apoptosis by blocking geranylgeranylation of the Rho family GTPase [12,14], resulting in disorganization of actin stress fibers [15]. Simvastatin was shown to foster enhanced expression of mutant p53 to down-regulate CD44 expression, therefore preventing breast cancer cell metastasis to bone [16]. Furthermore, simvastatin inactivates NF-κB, leading to de-repression of PTEN and repression of Bcl-xl to prevent breast cancer cell growth [17]. Notably, it was recently demonstrated that the mevalonate pathway is necessary and sufficient to maintain the malignant state of breast cancer cells in 3D culture [18]. However, specific antitumor targets and mechanisms of atorvastatin (AS) are poorly understood.

The Hippo pathway, with the transcriptional coactivator Yes-associated protein (YAP) as its downstream effector, is highly conserved throughout evolution [19]. The mammalian Hippo pathway consists of a core kinase cascade in which MST1/2 (the Hippo analog in *Drosophila*) phosphorylates the LATS1/2 kinases (Warts in Drosophila) [20]. Activated LATS subsequently phosphorylates YAP (at Ser127) and its paralog TAZ (at Ser89) (Yorkie in Drosophila), leading to inactivation of the transcriptional activity [21-23]. YAP promotes tumor metastasis through interacting with the TEAD/TEF transcription factors (Scalloped in Drosophila). Increased YAP/TEAD activity was observed in cancer progression and metastasis [24]. Upregulation of YAP and its nuclear localization strongly correlate with poor prognosis and tumor progression in multiple cancers, including breast [25], lung, colorectal, ovarian, and liver carcinomas [26]. Overexpression of YAP in a conditional YAP transgenic mouse model led to tissue overgrowth and tumorigenesis [27]. Together, these studies highlight a pivotal role of the Hippo–YAP pathway in cancer development and progression.

In this report, we demonstrated that atorvastatin or the geranylgeranyltransferase (GGTase) I inhibitor GGTI-298 inhibited proliferation of the estrogen receptor (ER)-negative breast cancer MDA-MB-231 cells and down-regulated PBK, indicating that PBK is a target gene of geranylgeranylation signaling. Consistent with the effect of atorvastatin or GGTI-298, knockdown of PBK or inhibition of PBK significantly impaired MDA-MB-231 cell proliferation. Furthermore, we found that knockdown of YAP down-regulated expression of PBK, suggesting that PBK is a target gene of YAP. Our studies therefore identified PBK as a down-stream effector of geranylgeranylation signaling and a target gene of YAP, and defined a PBK signaling pathway activated by geranylgeranylation and the Hippo signaling for breast cancer growth.

## Results

### Atorvastatin inhibited cell proliferation through inhibition of geranylgeranyl biosynthesis in ER-negtive (ER-) breast cancer cells

**Figure 1:**
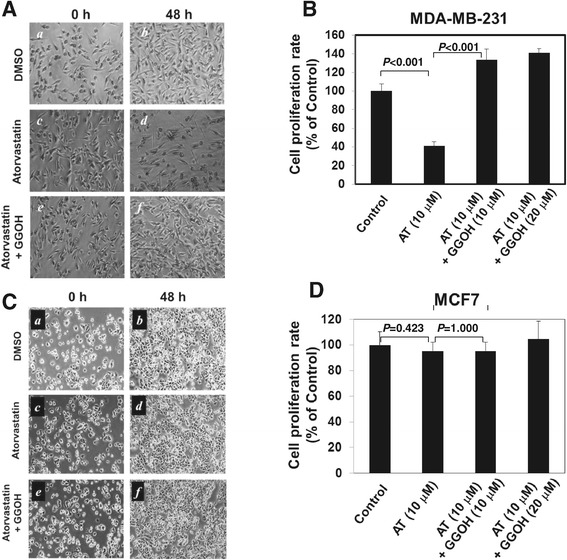
**Atorvastatin inhibited cell proliferation through inhibition of geranylgeranyl biosynthesis in MDA-MB-231, an ER-negtive (ER-) breast cancer cell line.** The ER- breast cancer MDA-MB-231 cells or the ER+ breast cancer MCF7 cells were treated with atorvastatin (10 μM) or atorvastatin (10 μM) plus GGOH (10 μM) for 48 hrs. The cells were photographed under a phase microscope and counted with a hemocytometer. The proliferation bar graphs were counted from three independent experiments. **A** and **B**, MDA-MB-231 cells; **C** and **D**, MCF7 cells.

To examine the effect of statins on breast cancer cell proliferation, we selected two breast cancer cell lines, the ER- cell line MDA-MB-231 and the ER+ cell line MCF7, in the experiments. Upon treated with atorvastatin for 48 hrs, proliferation of the ER- breast cancer MDA-MB-231 cells was significantly inhibited by about 60% (Figure 1A and B). Previous studies have shown that inhibition of geranylgeranyl biosynthesis is the cause for atorvastatin-induced cytotoxicity in breast cancer cells [28]. As shown in Figure 1A and B, addition of geranylgeraniol to the atorvastatin-treated cells rescued atorvastatin-caused inhibition of cell proliferation, confirming that impairing geranylgeranyl biosynthesis is the mechanism underlying the atorvastatin-induced cytotoxicity. Interestingly, atorvastatin had no significant effect on cell proliferation in the ER+ breast cancer cell line MCF7 (Figure 1C and D), suggesting that cell proliferation is independent of geranylgeranyl biosynthesis in MCF7 cells.

### Atorvastatin down-regulates expression of *PBK* in MDA-MB-231 cells but not MCF7 cells

**Figure 2:**
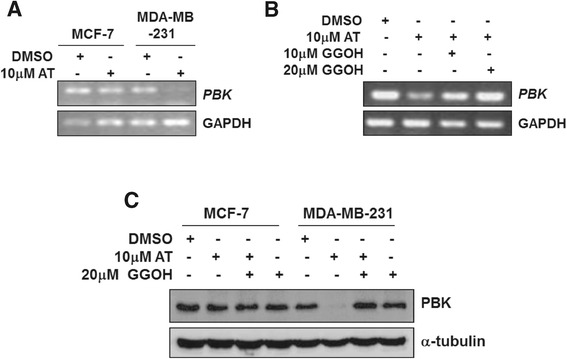
**Atorvastatin down-regulates expression of PBK in MDA-MB-231 cells but not MCF7 cells.** The cells were treated with atorvastatin (AT) or atorvastatin plus geranylgeraniol(GGOH) for 48 hrs. The effects of atorvastatin and GGOH on expression of *PBK* mRNA (in **A** and **B**) or PBK protein level (in **C**) were detected by RT-PCR and immunoblotting respectively.

As PBK is a biomarker for poor prognosis in breast cancer and plays a critical role in mitosis, we wonder if atorvastatin-induced cytotoxicity involving down-regulation of *PBK* gene expression. Consistent with the effect on the cell proliferation, atorvastatin treatment significantly down-regulated the mRNA level of *PBK* in the ER- breast cancer MDA-MB-231 cells, but not in MCF7 cells (Figure 2A). Adding geranylgeranyl to atorvastatin-treated MDA-MB-231 cells rescued down-regulation of *PBK* mRNA (Figure 2B). We further examined PBK protein level upon atorvastatin treatment and rescued by geranylgeranyl addition. As expected, PBK protein level was significantly reduced upon atorvastatin treatment and the reduction was rescued by geranylgeranyl addition in MDA-MB-231 cells (Figure 2C). Interestingly, although PBK protein level in MCF7 cells is comparable to that in MDA-MB-231 cells, it was not affected by atorvastatin treatment. Taken together, these data suggest that expression of PBK is tightly associated with geranylgeranyl-dependent cell proliferation in MDA-MB-231 cells, but not in MCF7 cells.

### Geranylgeranylation is required for expression of PBK in MDA-MB-231 cells

**Figure 3:**
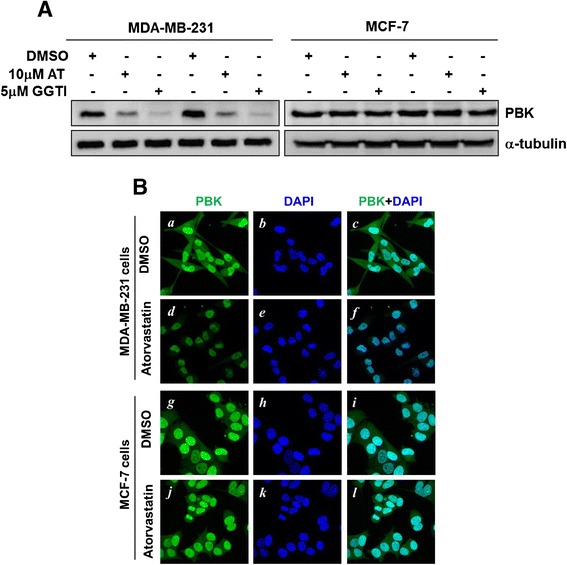
**Geranylgeranylation is required for expression of PBK in MDA-MB-231 cells. A**, The cells were treated with atorvastatin (AT) or the geranylgeranyltransferase I inhibitor GGTI-298 for 48 hrs. The effects of atorvastatin or GGTI-298 on PBK protein level were detected by immunoblotting. **B**, MDA-MB-231 or MCF7 cells were treated with DMSO (control) or atorvastatin for 48 hrs. The cells were stained with anti-PBK (green) or DAPI (blue).

Although our data indicate that inhibition of geranylgeranyl biosynthesis is the cause for atorvastatin-induced cytotoxicity and down-regulation of PBK expression in MDA-MB-231 cells, the biochemical pathway that is affected by inhibition of geranylgeranyl biosynthesis is not determined. Geranylgeranyl pyrophosphate (GGPP), which is the natural metabolite form of geranylgeranyl in mevalonate pathway, is the substrate of geranylgeranyltransferases used for protein geranylgeranylation. To test whether atorvastatin-induced down-regulation of PBK is through inhibition of geranylgeranylation, we treated MDA-MB-231 cells with the geranylgeranyltransferase I inhibitor GGTI-298 and examined PBK protein level in both MDA-MB-231 and MCF7 cell lysates. As shown in Figure 3A, GGTI-298 dramatically reduced protein level of PBK in MDA-MB-231 cells, but not in MCF7 cells, assayed by immunoblotting. Noticeably, the down-regulatory effect of GGTI-298 on PBK protein level was stronger than that of atorvastatin, probably due to incapability of atorvastatin to eliminate all the geranylgeranyl in the cells. To verify the down-regulation effect of atorvastatin on PBK expression in cells, we treated MDA-MB-231 and MCF7 cells with atorvastatin and monitored changes in the PBK protein level by immunofluorescent staining. As shown in Figure 3B, as expected, PBK is localized in nuclei, which is consistent with its function in nuclei for phosphorylation of histone H3 [10] and regulation of p53 tumor suppressor activity [8]. Treatment with atorvastatin caused a significant decrease in PBK staining in MDA-MB-231 cells (compare panel *d* with panel *a,* Figure 3B), while had no detectable effect on PBK staining in MCF7 cells (compare panel j with panel g, Figure 3B). Taken together, both immunoblotting and immunofluorescent staining data clearly indicate that geranylgeranylation controls *PBK* gene expression and PBK protein level in ER- breast cancer MDA-MB-231 cells, but not in ER+ breast cancer MCF7 cells.

### *PBK* is the YAP target gene in breast cancer MDA-MB-231 cells

**Figure 4:**
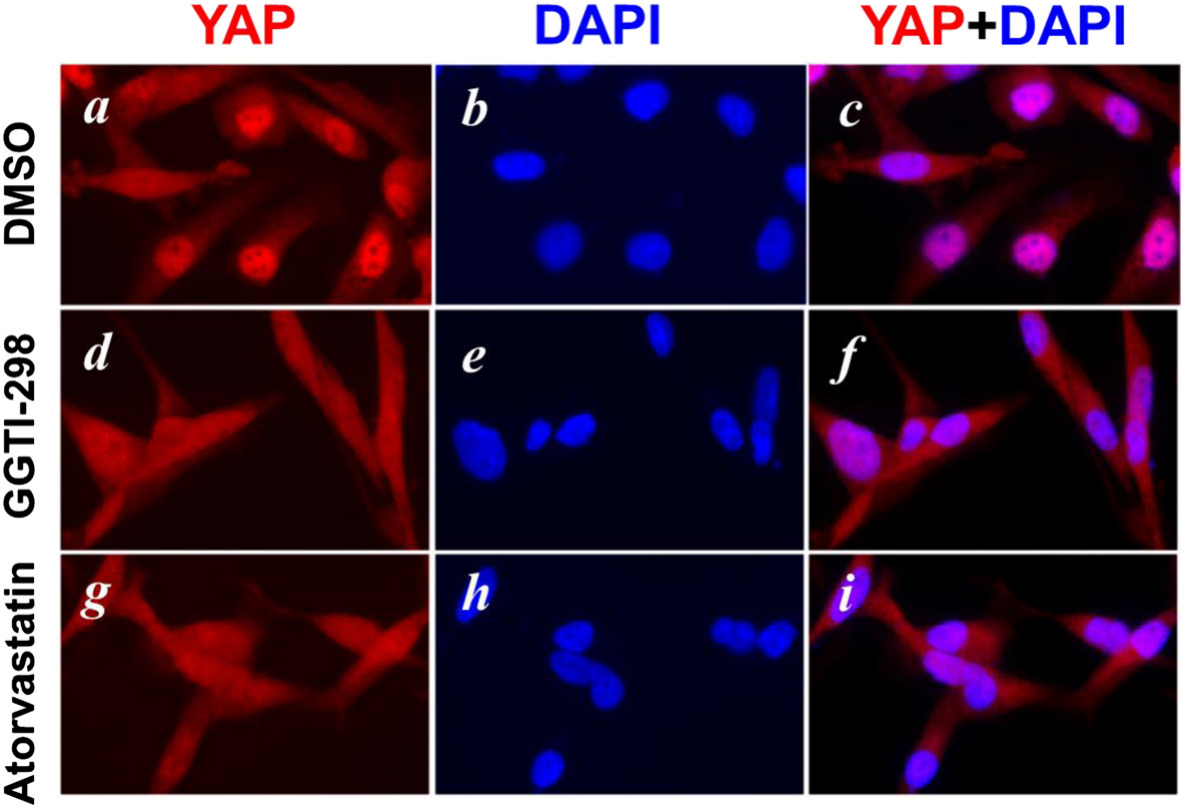
**Nuclear localization of YAP is inhibited by inhibition of Geranylgeranylation.** MDA-MB-231 cells were treated with atorvastatin **(panels d-f)** or the geranylgeranyltransferase I inhibitor GGTI-298 **(panels g-i)** for 48 hrs. The cells were stained with anti-YAP (red) **(panels a, d, and g)** or DAPI (blue) **(panels b, e, and h)**.

Several recent reports have shown that geranylgeranylation signaling is mediated by the Hippo-YAP/TAZ pathway for transcriptional activation and cancer cell proliferation and migration in MDA-MB-231 cells [28-30]. To confirm this, we treated MDA-MB-231 cells with GGTI-298 or atorvastatin for 48 hrs, and examined the effect on nuclear localization of YAP. Nuclear translocation is an activation process of YAP [28-30]. As shown in Figure 4, inhibition of geranylgeranylation by GGTI-298 or atorvastatin eliminated nuclear localization of YAP, indicating that YAP activation is dependent on geranylgeranylation signaling.

**Figure 5:**
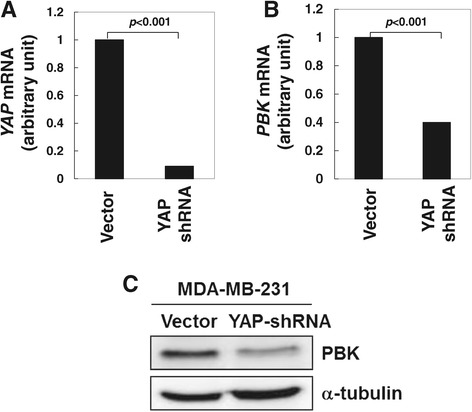
***PBK*** **is the YAP target gene in breast cancer MDA-MB-231 cells.** The YAP shRNA and the lentiviral vector pLKO.1 stable cell lines were established in MDA-MB-231 cells. The cells were cultured to 90% confluence for extraction of RNA and protein. *YAP* mRNA and *PBK* mRNA were detected by PT-PCR (in **A** and **B**). The PBK protein level was detected by immunoblotting of the cell lysates (in **C**).

We further examined whether the expression of *PBK* is dependent on YAP in MDA-MB-231 cells. As shown in Figure 5, knockdown of YAP by YAP shRNA significantly reduced both mRNA and protein levels of *PBK*, indicating that *PBK* is the target gene of YAP and suggesting that the geranylgeranylation signaling regulates PBK function through the YAP-mediated transcriptional activation.

### PBK is essential for proliferation of MDA-MB-231 cells

**Figure 6:**
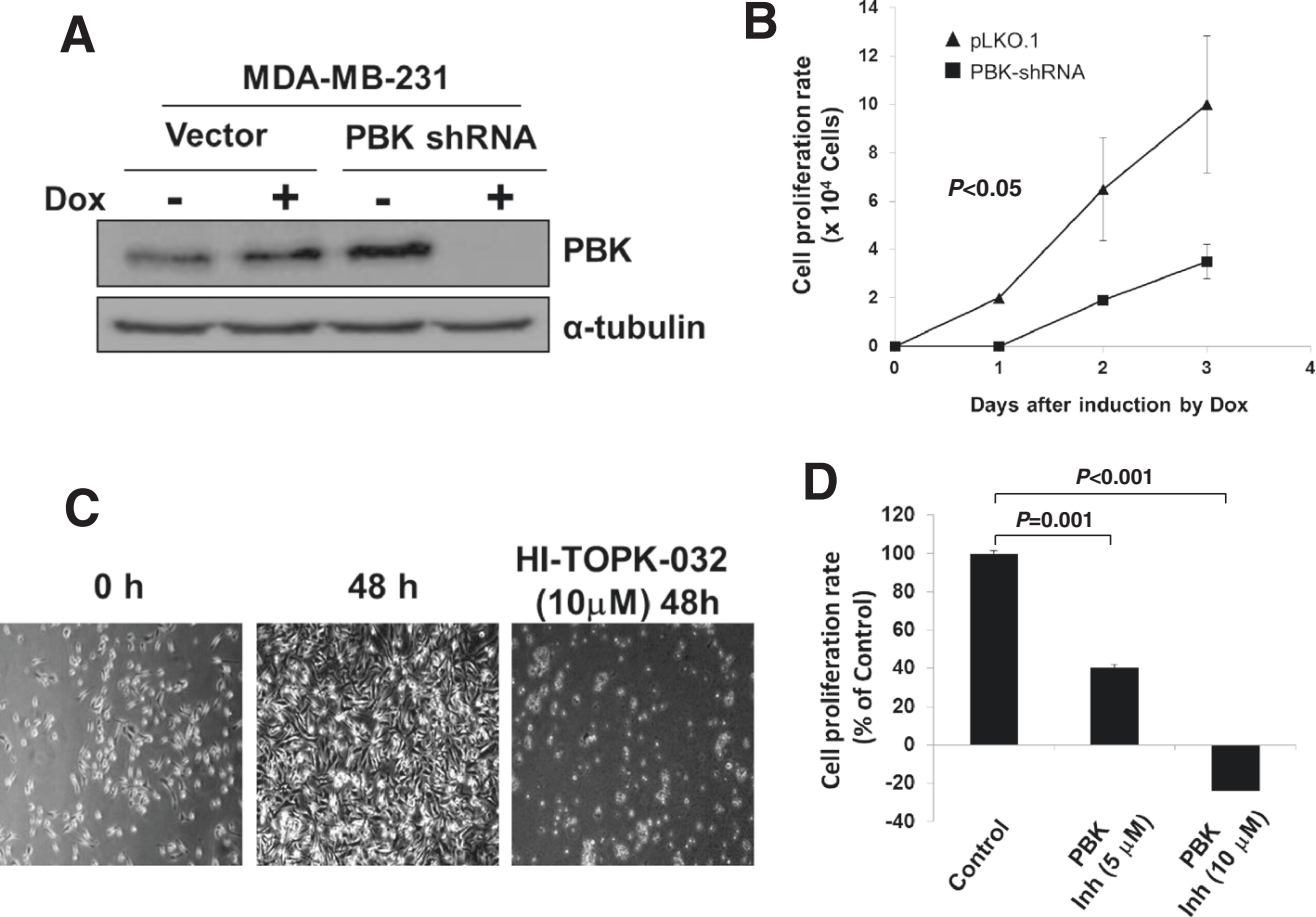
**PBK is essential for proliferation of MDA-MB-231 cells.** A and B, the PBK shRNA or the vector stable cell line was established in MDA-MB-231 cells using the tetracycline-induced lentiviral expression system. Expression of the shRNA was induced by doxycycline (1 μg/ml) for 3 days. The knockdown of PBK was detected by immunoblotting (in **A**), and effects of PBK knockdown on the cell proliferation was examined by cell counting with a hemacytometer (in **B**). Data are from at least three independent experiments. C and D, effects of the PBK inhibitor HI-TOPK-032 on MDA-MB-231 cell proliferation were observed and photographed under a phase microscope (in **C**) and the proliferation rate was quantified by cell counting with a hemacytometer (in **D**). The variation within the triplicate is indicated with the error bars. The proliferation rate is calculated using the formula PR = (Nt – No)/No, where PR stands for proliferation rate, No for the cell number at seeding, and Nt for the cell number at t time point after seeding.

To determine whether PBK mediates the geranylgeranylation signaling in breast cancer cell proliferation, we examined the effect of PBK knockdown and the kinase inhibition on MDA-MB-231 cell proliferation. As shown in Figure 6A and B, depletion of PBK by PBK shRNA in MDA-MB-231 cells significantly inhibited cell proliferation, indicating an important role of PBK in the cancer cell proliferation. To confirm the PBK knockdown effect, we employed the PBK kinase inhibitor HI-TOPK-032 to treat MDA-MB-231 cells for two days and monitored the effect on cell proliferation. As shown in Figure 6C and D, inhibition of PBK kinase activity in MDA-MB-231 cells by 10 μM HI-TOPK-032 caused drastic cell death. These data suggest that PBK is the protein mediating the geranygeranylation signaling in MDA-MB-231 cell proliferation. Interestingly, we also observed that the PBK inhibitor HI-TOPK-032 significantly inhibited proliferation of MCF7 cells (data not shown), suggesting that PBK also plays an important role in proliferation in MCF7 cells. However, the connection of geranylgeranylation signaling to expression of PBK is missing in MCF7 cells (Figure 3). These data demonstrate that regulation of expression, not activity, of PBK is the mechanism underlying geranylgeranylation signaling-mediated breast cancer cell proliferation.

## Discussion

Breast cancer is the leading cause of cancer death in females worldwide. Unraveling the molecular and cellular mechanisms underlying breast cancer progression and metastasis is important for development of targeting drugs for breast cancer therapy. In this report, we have shown that atorvastatin inhibits breast cancer cell proliferation through impairing geranylgeranylation. Our data indicate that geranylgeranylation signaling controls expression of *PBK*, a gene whose product regulates cell mitosis, thus is important for cancer cell proliferation. Furthermore, we found that *PBK* is a target gene of YAP, a transcription co-activator in the Hippo pathway, suggesting that geranylgeranylation signaling activates YAP to regulate expression of *PBK*. Finally, we have shown that PBK is essential for breast cancer cell proliferation. However, expression of PBK is responsive to geranylgeranylation signaling only in ER- breast cancer MDA-MB-231 cells, not in ER+ breast cancer MCF7 cells. This observation raises a possibility that linkage of geranylgeranylation signaling to expression of PBK is established during breast cancer progression.

Our findings are consistent with recent studies that discovered the role of geranylgeranylation signaling in regulating the Hippo-YAP/TAZ pathway for breast cancer cell proliferation and migration [28]. The geranylgeranylation signaling interplays with the Hippo pathway in regulating YAP/TAZ transcriptional activity. Currently the geranylgeranylated proteins that transduce signaling to the Hippo-YAP/TAZ pathway have not been identified. Prenylation, including farnesylation and geranylgeranylation, is essential for proper localization and activity of the RAS superfamily of small GTPases and heterotrimeric G-protein gamma subunit. The Rho GTPase subfamily is known to be geranylgeranylated and closely linked to cancer progression [31]. Particularly, RhoA are thought to play a role in cell proliferation. Recent studies have shown that GPCR signaling activates RhoA GTPase that leads to inhibition of the Hippo kinases and activation of YAP/TAZ [28,32]. We speculate that Rho GTPase might be the geranylgeranylated protein signaling to the Hippo pathway to activate YAP and expression of *PBK*.

Atorvastatin is one of the most popular medicines for reduction of cholesterol. The anti-breast cancer effect of atorvastatin has a huge impact on prevention of breast cancer. Our studies provide fundamental knowledge to help clinicians to use atorvastatin in breast cancer therapy and prevention. Our data suggest that atorvastatin can induce cytotoxicity only to certain type of breast cancer cells. The breast cancer cell must have a signaling context of geranylgeranylation signaling and the Hippo-YAP/TAZ pathway that confers the effect of atorvastatin on down-regulation of PBK. In addition, establishment of linkage of geranylgeranylation signaling to the Hippo-YAP/TAZ pathway in breast cancer cells seems developed in breast cancer progression, because our recent studies found that ER- breast cancer cells are more sensitive to atorvastatin than ER+ breast cancer cells [28]. Thus, statins might be an effective anti-cancer drug for advanced stage breast cancer. We will further investigate the role of geranylgeranylation signaling in promoting cancer progression and validate geranylgeranylation signaling as a key targeting pathway in advanced breast cancer therapy.

PBK has been identified as a kinase regulating mitosis by phosphorylation of GPSM2 (G-protein signaling modulator 2) in breast cancer cells [33]. PBK also interacts with p53 to regulate expression of cell cycle genes [8]. Multiple studies have found that PBK overexpresses in breast, prostate, colon, bladder, and lung cancers and is a prognostic biomarker for poor outcomes [2-5]. Previous studies have shown that expression of PBK is regulated by Myc and E2F1 transcriptional factors [34]. Immunofluorescent staining in Figure 3B has shown that PBK is localized in nuclei, confirming that PBK is a nuclear kinase and functions in phosphorylation of nuclear proteins. Furthermore, our results indicate that PBK is a target gene of YAP (Figure 5). Interestingly, it has been observed that E2F1 is also a target gene of YAP [35]. These studies suggest that E2F1 may mediate YAP-activated expression of PBK in MDA-MB-231 cells. Furthermore, our studies have shown that PBK is connected to geranylgeranylation signaling most likely in advanced stage breast cancer, and essential for breast cancer cell proliferation, confirming that PBK is an important molecular target for breast cancer therapy. Thus, down-regulation and inhibition of PBK by either targeting geranylgeranylation signaling and the Hippo-YAP/TAZ pathway or directly impairing the kinase activity are promising approaches for breast cancer therapy, particularly advanced stage breast cancer therapy.

## Conclusions

Inhibition of geranylgeranyl biosynthesis by atorvastatin or geranylgeranyltransferase I by GGTI-298 significantly reduced cell proliferation of the ER-negative breast cancer MDA-MB-231 cells and down-regulated expression of PBK in ER-negative breast cancer MDA-MB-231 cells. The geranylgeranylation-dependent cell proliferation is correlated with geranylgeranylation-dependent PBK expression. Knockdown of YAP also down-regulated expression of PBK, indicating that PBK is a target gene of YAP and suggesting that YAP may mediate geranylgeranylation-dependent expression of PBK. Direct knockdown of PBK by shRNA or inhibition of PBK activity by its kinase inhibitor severely impaired breast cancer cell proliferation. Taken together, we conclude that PBK mediates geranylgeranylation signaling-promoted breast cancer cell proliferation.

## Materials and methods

### Materials

Geranylgeraniol (G3278), GGTI-298 (G5169) and HI-TOPK-032 (SML0796) were purchased from Sigma-Aldrich. Atorvastatin calcium was from WuXi Sigma. Anti- PBK/TOPK (C-term) antibody (ab75987) was from Abcam, anti-Tubulin (G436) from Bioworld, and anti-β-actin (ac-15) from Sigma.

### Cell culture

Human kidney cell line HEK293T and human breast cancer cell lines MCF-7 and MDA-MB-231 were grown in Dulbecco modified Eagle Medium (DMEM) medium(HyClone)supplemented with 10% fetal bovine serum (Excell bio), 100 U/mL penicillin, and 100 mg/mL streptomycin in 5% CO2 at 37°C. Transfection of plasmids was performed with Lipofectin transfection reagent according to the manufacturer’s protocol.

### Reverse transcription PCR (RT-PCR) analysis

Total RNA was extracted using RNAiso Plus (TaKaRa) kit and reverse-transcribed into cDNA by RevertAid First Strand cDNA Synthesis Kit (TaKaRa) according to the manufacturer’s instructions. Quantitative PCR analysis was performed using iTaq Universal SYBR Green supermix (2×) (BIO-RAD). The primer pairs used for quantitative PCR are: PBK (human) forward primer: 5′-CCTTTGGCCTTACTTTGTG -3′; PBK (human) reverse primer: 5′-ACGATCTTTAGGGTCTTCAT-3′; GAPDH (human) forward primer: 5′-AACGGATTTGGTCGTATTG-3′; GAPDH (human) reverse primer: 5′- GGAAGATGGTGATGGGGAT -3′.

### Preparation of cell lysates and immunoblotting

Culture medium was removed and cells were washed with cold PBS and lysed in precooled mammalian cell lysis buffer (40 mM HEPES, pH 7.4, 1% Triton X-100, 100 mM NaCl, 1 mM EDTA, 25 mM Beta-Glycerolphosphate, 1 mM Na-orthovanadate, 10ug/ml Leupeptin and 10ug/ml Aprotinin). The SDS-PAGE samples were prepared by addition of 5 × SDS sample buffer directly to the lysates, followed by rigorous vortex and denatured at 100°C for 10 min. Electrophoresis was run on 10% NuPAGE Bis-Tris SDS gels, and separated proteins were transferred onto Immobilon PVDF-FL (Millipore) membranes. The membranes were incubated with primary antibodies overnight at 4°C, followed by incubating with secondary antibodies for 1 h at room temperature. The protein bands were visualized by Enhanced Chemiluminescence Plus reagent (Millipore). The density of the bands was quantified using Quantity One software (MiNiCHEMI).

### Immunofluorescent staining

The cells were cultured in glass coverslip-bottomed culture dishes (MatTek, Ashland, MA) to 50-80% confluence. After the culture medium was aspirated, the cells were rinsed with PBS twice, fixed with 3.7% paraformaldehyde at 25°C for 30 min, and permeabilized with 0.2% Triton X-100 in PBS at 25°C for 20 min. After washing with TBST, the cells were incubated with primary antibody at 37°C for 1 h. Then the cells were washed with TBST three times and incubated with secondary antibody that was conjugated with a fluorescent dye at 37°C for 1 h. Finally, the cells were washed with TBST three times, and the immunofluorescence staining was visualized under a Nikon inverted fluorescent microscope. The nuclei were stained with DAPI.

### Lentiviral shRNA cloning, production, and infection

Knockdown of PBK or YAP was carried out by infection of cells with lentiviral vector-loaded shPBK or shYAP. The PBK shRNA target sequence is CTCTTCTCTGTATGCACTAAT; and the YAP shRNA targeting sequences is CCCAGTTAAATGTTCACCAAT. To produce the lentiviral particles, the pLKO. 1-tet-puro vector was co-transfected with the packaging plasmids psPAX2 and pMD2.G into HEK 293 T cells, and the cultured supernatant containing the viral particles was collected at 24, 48 and 72 hrs after transfection. This supernatant was used as the shRNA viral stock solution. For lentiviral infection, the shRNA viral stock solution was added into MDA-MB-231 cell culture medium for 24 hrs in the presence of 4 μg/mL polybrene. In general, the viral infection efficiency was about 80%. After the infection, cells were cultured in DMEM plus 10% FBS and 2 μg/ml puromycin. Selection of puromycin-resistant cell colonies was carried out 72 h after transfection. The cell colonies resistant to puromycin were selected and cultured in DMEM plus 10% FBS, 1 μg/ml tetracycline and 2 μg/ml puromycin. The knockdown efficiency was evaluated by detection of protein level with immunoblotting or mRNA level with qRT-PCR.

### Statistical analysis

The experimental data are analyzed statistically using Student’s *t*-test for two-treatment comparisons. *P* < 0.05 is considered as significant.

## Abbreviations

ER: Estrogen receptor
GGOH: Geranylgeraniol
GGTI: Geranylgeranyltransferase inhibitor
HMG-CoA: Hydroxymethylglutaryl co-enzyme A
PBK: PDZ-binding kinase
TOPK: T-lymphokine-activated killer cell-originated protein kinase
YAP: Yes-associated protein
